# Perfectionism and Eating Behavior in the COVID-19 Pandemic

**DOI:** 10.3389/fpsyg.2021.580943

**Published:** 2021-06-03

**Authors:** Mariacarolina Vacca, Alessandra De Maria, Luca Mallia, Caterina Lombardo

**Affiliations:** ^1^Department of Psychology, Sapienza University of Rome, Rome, Italy; ^2^Department of Movement, Human and Health Sciences, University of Rome “Foro Italico”, Rome, Italy

**Keywords:** COVID-19, perfectionism, multidimensional, mediation, eating behavior, stress

## Abstract

The novel coronavirus disease 2019 (COVID-19) represents a massive global health crisis leading to different reactions in people. Those reactions may be adaptive or not depending on situational or psychological processes. Disordered eating attitudes and behaviors are likely to be exacerbated by the pandemic through multiple pathways as suggested by Rodgers et al. (2020). Among the psychological variables that may have increased dysfunctional eating attitudes and behaviors as a consequence of the social distancing and isolation, we looked at perfectionism. Perfectionism is a well-recognized risk and maintaining factor of eating-related symptoms and interact with stress increasing the probability of dysfunctional reactions (e.g., Wang and Li, 2017). The present study investigated the relationship between multidimensional perfectionism and eating behaviors by considering the mediating role of psychological distress. Data were collected from two countries (Italy and Spain) by means of an online survey. The samples included 465 (63.4% female) participants from Italy and 352 (68.5% female) from Spain. Participants completed the short form of the Hewitt and Flett Multidimensional Perfectionism Scale (Lombardo et al., 2021) to assess self-oriented, other-oriented and socially prescribed perfectionism, as well as the short form of Three Factors Eating Questionnaire (Karlsson et al., 2000) and the Italian version of Depression Anxiety and Stress Scale-21 (Bottesi et al., 2015), respectively used to assess restrictive, emotional and uncontrolled eating on one hand, and depression, anxiety and stress on the other. Multigroup analysis was performed to test the hypothesized model. Results showed that other-oriented and socially prescribed perfectionism were indirectly related to most of the dysfunctional eating aspects through the mediation of psychological distress, and the pattern obtained was consistent in both countries. These findings evidence that the psychological distress potentially related to the COVID-19 disease mediates the negative impact of interpersonal perfectionism and the tendency to eat in response to negative emotions.

## Introduction

The novel coronavirus disease 2019 pandemic (COVID-19; World Health Organization [WHO], 2020) has spread to most countries in the world and represents a massive global health crisis. To date, the number of COVID-19 patients has increased dramatically, with 4,320,946 currently positive cases in the world. Moreover, as suggested by the CDC^1^, governors of most countries ask to all citizens to adopt social distancing, quarantine and isolation as strategies for containment. According to the Stress Theory (Norris et al., 2002), public emergencies may trigger negative emotions and enhance dysfunctional cognitive beliefs, predisposing people to mental health difficulties. The COVID-19 pandemic is one of the most stressful situations for its own unpredictability and prolonged social isolation, therefore, it is crucial to understand the potential psychological outcomes influenced by this health emergency. Different reactions of people to the COVID-19 pandemic may involve their personality, as dispositional traits may shed light on people’s different reactions (Blagov, 2020). Findings from personality research have highlighted that personality-related variables could profoundly change the impact of stress in challenging situations (Greene et al., 2020). Personality traits play an essential role in predicting coping strategies with emotional distress in stressful events and have significant consequences on mental health (Kessler, 1997).

One of the personality dimensions potentially implicated is perfectionism, a multidimensional personality characteristic composed by two major dimensions, namely perfectionistic strivings (i.e., incessantly demanding perfection of oneself) and perfectionistic concerns (i.e., extreme concern over mistakes and others’ evaluations; see Stoeber and Otto, 2006). These two dimensions respectively reflect adaptive and maladaptive facets of perfectionism since they showed opposite associations with psychological adjustment and well-being (see Limburg et al., 2017, for a review). Hewitt and Flett (1991) developed a multidimensional model of perfectionism that distinguishes inter and intrapersonal facets reflecting both perfectionistic strivings and perfectionistic concerns. According to this model, self-oriented perfectionism (SOP), a key aspect of perfectionistic strivings, refers to the tendency to set high standards and the belief that striving for perfection is personally crucial. Socially prescribed perfectionism (SPP) refers to the perception that other people place unrealistic expectations for oneself and reflects perfectionistic concerns. Other oriented perfectionism (OOP) involves the tendency to expect that others should achieve unrealistic outcomes (Hewitt and Flett, 1991), and is typically conceptualized as a component of perfectionistic concerns (see Limburg et al., 2017), although some authors suggested it should be considered as a unique distinct form (e.g., Sherry et al., 2016). These aspects of perfectionism show different associations with mental well-being and maladjustment. More specifically, SPP is a maladaptive form of perfectionism as it results significantly related to negative characteristics and psychological distress (e.g., Stoeber and Yang, 2010). OOP is an ambivalent form of perfectionism, sometimes associated with positive, sometimes with dysfunctional outcomes (Stoeber, 2012). On the other hand, SOP has been proposed as the adaptive side of perfectionism (Stoeber and Otto, 2006), more consistently associated with functional outcomes (Lee et al., 2012), although its adaptive nature is still debated (Molnar et al., 2012).

Perfectionism increases concerns about under achievements, especially in stressful situations (Hasel and Besharat, 2011), as it plays an important role in modifying psychophysiological responses to psychosocial or environmental stress. Research has also shown that individuals with perfectionism display higher levels of distress than non-perfectionists and use ineffective strategies to cope with life challenges (Dunkley et al., 2003; Wagner, 2016). Their greater perception of stress is generated by the pursuit of unrealistic standards which often end in failure (Flett et al., 2020).

Studies available in literature employing the Hewitt and Flett (1991) model showed mixed findings concerning the relationship between perfectionistic facets and perceived stress. Some authors found positive associations between perceived stress and SOP and SPP (Molnar et al., 2012) and non-significant results concerning OOP (Smith et al., 2017). However, in some cases, positive correlations between OOP and perceived stress were observed (Chang and Rand, 2000). Meta-analytic evidence on the associations between perfectionism and general psychological distress indicated larger effects for perfectionistic concerns (e.g., SPP) relative to perfectionistic strivings (e.g., SOP), thus confirming the dual nature of the construct in explaining perceived stress (Limburg et al., 2017).

When people with high perfectionism perceive stress, they are also more prone to report psychopathological difficulties like anxiety (see Burgess and Di Bartolo, 2016), depression (Flett et al., 2016), and eating disorders (e.g., Mello, 2016).

Perfectionistic individuals are more likely to develop maladaptive eating attitudes (Machado et al., 2014) with evidence showing that both the major dimensions discussed above (i.e., perfectionistic strivings, perfectionistic concerns) equally contribute to explaining variance in dysfunctional eating outcomes (Limburg et al., 2017). It was observed that the association between perfectionistic dimensions and eating symptoms increased in magnitude under stress situations (Ruggiero et al., 2003; Sassaroli and Ruggiero, 2005). Moreover, findings showed that stress triggers maladaptive eating behaviors in individuals with high perfectionistic concerns, thus suggesting that the mechanism underlying the association between perfectionism and eating symptoms may be related to stress. This issue was addressed in a cross-sectional study examining the mediating role of stressful life events in the relationship between self-evaluative perfectionism (i.e., maladaptive form of perfectionism) and eating disorder symptoms (Mello, 2016). Results show that stress partially mediates this association. More specifically, high self-evaluative perfectionism was associated with high perceived stress, that, in turn, explained significant variance in eating symptoms. A more recent work proposing a mediation model tested the impacts of adaptive and maladaptive facets of perfectionism on emotional eating through perceived stress (Wang and Li, 2017). Emotional eating consists of the propensity for eating in response to negative emotions (Hawks et al., 2003), and resulted to be high among individuals who experienced stress (Tan and Chow, 2014). Authors found that maladaptive perfectionism was positively associated with stress, which in turn aggravated emotional eating behavior. Differently, the indirect effect of adaptive perfectionism on emotional eating through stress was significantly negative, suggesting that adaptive perfectionists are less vulnerable to emotional eating even when stressed. Taken together, these findings suggest that stress may be crucial to understand the complex relationship between perfectionism and eating-related symptoms.

In this theoretical framework, the present study aimed to investigate whether psychological distress during the COVID-19 pandemic mediates the relationship between perfectionistic aspects and dysfunctional eating behaviors. More specifically, three cognitive and behavioral components of eating were analyzed, namely cognitive restraint (i.e., the tendency to consciously restrict food intake), uncontrolled eating (i.e., overeating after being exposed to food cues) and emotional eating (i.e., the propensity to eat in response to negative emotions). Individuals rigidly engaging in dietary restraint by limiting food/calories often experience higher disinhibition that in turn might lead to losing control resulting in overeating and subjective feelings of food craving (Keränen, 2011). These three eating behaviors resulted to be closely related to psychological distress as normally the perception of stress can facilitate unhealthy eating in people (e.g., Richardson et al., 2015).

The proposed model was tested in Italy and Spain, the two main European countries most affected by the 2019 coronavirus disease, reporting respectively more than 105,000 and 94,000 total confirmed cases as of April 1st, 2020 (World Health Organization [WHO], 2020). Both countries have faced similar outbreaks since the beginning of the infection spread, showing a rapid increase in both positive cases and deaths compared to other European countries (Giangreco, 2020). Indeed, Spain suffered a surge in the pandemic within a few days which forced the country to follow Italy in the exceptional prevention measures, thus implementing quarantine in less than a week away.

Concurrently, previous research on personality has also provided evidence to support the presence of cultural differences in the correlates of perfectionism (e.g., Francisco et al., 2015), as well as in latent mean scores (e.g., Pietrabissa et al., 2020). Among these, the study carried out by Francisco et al. (2015) has demonstrated that the direct and indirect role of perfectionism on body dissatisfaction varies between Portuguese and Spanish adolescents, despite belonging to two neighboring countries of the southern Europe. In addition, a very recent study reported significantly lower latent factor mean of self-oriented and socially prescribed perfectionism in Italians than in Spaniards (Pietrabissa et al., 2020).

Although Italy and Spain share similar lifestyles, cultural heritages and religious and family values that are less marked, or not present, in other European countries (Micheli, 2012), they remain distinct countries. For example, Italy exhibited an individualistic tendency focused more on competition, results and success rather than on quality of life, unlike Spain (Hofstede et al., 2005). This difference has displayed an important effect on the predictive role of the two dimensions of perfectionism on various psychological outcomes (e.g., Stoeber et al., 2013). Therefore, in addition to clarifying the role of psychological distress due to the COVID-19 pandemic, an attempt was made to investigate possible cross-cultural differences in the proposed model considering two distinct, albeit culturally similar, countries.

It was hypothesized that the three perfectionistic components derived from the Hewitt and Flett’s (1991) model would be related to psychological distress that, in turn, would impact eating behaviors. The effect of perfectionistic dimensions (i.e., SOP, SPP, OOP) on psychological distress was estimated to be different according to the specific dimension analyzed. Previous evidence suggested that these aspects of perfectionism showed different associations with indices of stress and evidence is mixed. For instance, SPP resulted to be consistently positively related to perceived stress (e.g., Smith et al., 2017). Results on SOP revealed non-significant associations with stress in some cases (e.g., Flett et al., 2016), otherwise some authors found that high SOP predicts high distress (Molnar et al., 2012). Evidence on OOP suggested that it did not play a significant role in stress (e.g., Aryani and Koesma, 2020) although other studies showed significant positive OOP-stress associations (Han and Park, 2019).

Basing on these findings, the present study aims to get some additional insights for answering the following questions:

- Which dimensions of perfectionism are related to psychological distress? In other words, considering the relative adaptiveness of SOP and OOP continues to be controversial (e.g., Molnar et al., 2012; Han and Park, 2019; Aryani and Koesma, 2020) should these dimensions be considered adaptive or maladaptive?
- Does psychological distress (i.e., stress experienced during the COVID-19 pandemic) mediate the relationship between perfectionistic aspects and disordered eating attitudes and behaviors, as indicated by previous evidence (e.g., Wang and Li, 2017)?
- Which dimensions of perfectionism are related to disordered eating attitudes and behaviors when we take into account the psychological distress? Systematic evidence shows that both perfectionistic concerns (i.e., SPP, OOP) and perfectionistic strivings (i.e., SOP) are relatively equally related to eating disturbances (see Limburg et al., 2017) but it is not clear whether this effect is direct or is fully mediated by other relevant variables like the impact of stress.
- Are there key cross-cultural differences in any of the associations described above in two distinct national cultures (Italy versus Spain)?

## Materials and Methods

### Participants

The study involved three samples of participants: two recruited in Italy from April 26th to May 2nd 2020, and one recruited in Spain from April 26th to May 9th 2020. The first Italian sample (i.e., *psychometric sample*) included 360 participants (42.5% male; mean = 22.99 years; *SD*: 5.76; range: 18–77) recruited with the aim to validate the Italian short version of the Three-Factor Eating Questionnaire (TFEQ; Karlsson et al., 2000). The remaining main samples were recruited from Italy and Spain in order to test the hypothesized model and invariance across countries. The Italian main sample comprised 465 participants (35.9% male; mean = 36.76 years; *SD*: 12.86; range: 18–72), of which the 0.4% was tested and resulted positive for COVID-19 and the 6% was subjected to special restrictions related to their health (e.g., mandatory quarantine). The Spanish main sample comprised 352 participants (31.5% male; mean = 38.05 years; *SD*:13.96; range: 18–71), of which the 1.1% was tested and resulted positive for COVID-19 and the 14.5% was subjected to restrictions related to their health. At the time of the survey, Italy had imposed a national lockdown for about 7 weeks, while Spain had set the lockdown for about 6 weeks. A greater portion of the Italian main sample reported to respect every day the provided restrictions (80.6%), compared to the Spanish one (74.7%). As detailed in Table 1, the two main samples resulted statistically different only on the education level (χ^2^ = 66.15; *p* < 0.001), while no differences emerged on age [*F*_(__1_,_815__)_ = 1.89; *p* = 0.17], gender composition (χ^2^ = 4.17; *p* = 0.12), marital status (χ^2^ = 7.98; *p* = 0.16), family income (χ^2^ = 0.64; *p* = 0.89), as well as on Body Mass Index [*F*_(__1_,_815__)_ = 1.67; *p* = 0.2].

**TABLE 1:** Socio-demographic characteristics of the samples.

Characteristics	Italian psychometric Sample (*n* = 360) Mean (*SD*) Frequency (%)	Main samples		Main samples comparison	
		Italy (*n* = 465) Mean (*SD*) Frequency (%)	Spain (*n* = 352) Mean (*SD*) Frequency (%)	*F* χ^2^	*P*
Age (years)	22.99 (5.76)	36.76 (12.86)	38.05 (13.96)	1.89	0.169
**Gender**				4.17	0.124
Male	153 (42.5%)	167 (35.9%)	111 (31.5%)		
Female	207 (57.5%)	295 (63.4%)	241 (68.5%)		
**Marital status**				7.98	0.157
Married/Cohabiting	24 (6.7%)	209 (44.9%)	143 (40.6%)		
Separated	/	8 (1.7%)	5 (1.4%)		
Divorced	/	9 (1.9%)	18 (5.1%)		
Widowed	/	6 (1.3%)	5 (1.4%)		
Never married	336 (93.3%)	232 (49.9%)	181 (51.4%)		
Other	/	1 (0.2%)	/		
**Level of education**				66.15	<0.001
Primary school	/	/	8 (2.3%)		
Lower secondary school	3 (0.8%)	17 (3.7%)	23 (6.5%)		
Upper secondary school	267 (74.2%)	144 (31%)	49 (13.9%)		
Undergraduate/Master	76 (21.1%)	222 (47.7%)	150 (42.6%)		
Ph.D. Scholar/Specialization	12 (3.3%)	78 (16.8%)	107 (30.4%)		
Other	2 (0.6%)	4 (0.9%)	15 (4.3%)		
**Family income**				0.638	0.888
Very low	5 (1.4%)	8 (1.7%)	5 (1.4%)		
Low	44 (12.2%)	70 (15.1%)	51 (14.5%)		
Middle	280 (77.8%)	340 (73.1%)	265 (75.3%)		
High	31 (8.6%)	47 (10.1%)	31 (8.8%)		
Very high	/	/	/		
**Body mass index (kg/m^2^)**	22.41 (3.13)	23.74 (4.19)	25.02 (20.80)	1.67	0.196

### Procedure

All participants were contacted through a non-random convenience recruitment procedure: information related to the study and the link for filling the questionnaires in were spread through acquaintances, word of mouth and social media. The Italian-language version of the survey was identical in content to the Spanish-language version, and both were hosted on the same secure Internet-based survey-hosting platform (i.e., Survey Monkey). Participants were required to indicate agreement with the informed consent document explaining the purpose of the study and highlighting the ethical principles (i.e., confidentiality of information, voluntary participation, withdrawal from participation at any time) before they could enter the survey. Participants were eligible to participate if: (1) they were living either in Italy or Spain at the time of the survey, and (2) they aged 18 or more years old. These inclusion criteria were set to ensure that participants adequately represented the two cultures understudy during the pandemic period. This study was conducted in accordance with the Helsinki Declaration and received approval from the Institutional Review Board of the Department of Psychology, Sapienza University of Rome.

### Instruments

All the respondents filled out the online questionnaire measuring the following key variables in Italian and Spanish language, respectively for both Italian samples and the Spanish sample.

#### Socio-Demographic Characteristics

The questionnaire assessed participants’ age, gender, marital status, education, family income, and information related to the COVID-19 pandemic.

#### Multidimensional Perfectionism

Participants’ perfectionism was assessed using the short version of the Hewitt and Flett Multidimensional Perfectionism Scale (HFMPS; Hewitt et al., 2008), consisting of 15 items, 5 items for each dimension, namely *self-oriented perfectionism* (SOP; e.g., “One of my goals is to be perfect in everything I do”), *socially prescribed perfectionism* (SPP; e.g., “The better I do, the better I am expected to do”) and *other-oriented perfectionism* (OOP; e.g., “I have high expectations for the people who are important to me”). Items were rated using a 7-point Likert scale ranging from 1 (disagree) to 7 (agree), with higher scores indicating greater perfectionism. This brief version has previously validated for use in Italy (Lombardo et al., 2021), whereas for the Spanish sample we used the corresponding 15 items^2^ from a previously validated Spanish long version of the scale (see Rodríguez Campayo et al., 2009). The reliability coefficients (i.e., Cronbach’s Alpha) of the three HFMPS’s sub-scales across the two main samples are reported in Table 2.

**TABLE 2:** Reliability coefficients and descriptive of the Key Measures across the Italian and Spanish main samples.

	Cronbach’s α		Mean (*SD*)		*F*	*P*	Partial Eta Squared
	Italy	Spain	Italy	Spain			
**Multidimensional perfectionism**							
Self-oriented perfectionism (SOP)	0.84	0.89	4.76 (1.25)	4.84 (1.29)	0.71	0.398	0.001
Other-oriented perfectionism (OOP)	0.75	0.77	4.18 (1.19)	4.05 (1.19)	2.25	0.134	0.003
Socially prescribed perfectionism (SPP)	0.73	0.75	3.81 (1.25)	3.52 (1.20)	10.87	0.001	0.013
**Psychological distress**							
Depression	0.89	0.86	0.82 (0.62)	0.62 (0.58)	21.58	<0.001	0.026
Anxiety	0.84	0.83	0.47 (0.49)	0.47 (0.54)	0.07	0.796	0.000
Stress	0.89	0.89	1.11 (0.62)	0.94 (0.69)	13.84	<0.001	0.017
**Eating behaviors**							
Emotional eating	0.85	0.85	2.12(0.83)	2.05(0.84)	1.44	0.230	0.002
Uncontrolled eating	0.86	0.90	2.05(0.60)	2.15(0.68)	4.80	0.029	0.006
Cognitive restrain	0.84	0.83	2.33(0.70)	2.28 (0.69)	0.95	0.331	0.001

#### Psychological Distress

The respondents’ psychological distress was assessed using the Depression Anxiety Stress Scales (DASS; Lovibond and Lovibond, 1995), consisting of 21 items measuring three different aspects, namely *depression* (seven items, e.g., “I couldn’t seem to experience any positive feeling at all”), *anxiety* (seven items, e.g., “I was aware of dryness of my mouth”), and *stress* (seven items, e.g., “I found it hard to wind down”). Respondents read statements about these constructs and record their answers using a 4-point Likert-type scale ranging from 0 (Did not apply to me at all) to 3 (Applied to me very much or most of the time). Past studies in Italy (Bottesi et al., 2015) and Spain (Daza et al., 2002) have attested the validity and the reliability of the scale. The reliability coefficients (i.e., Cronbach’s α) of the three DASS’s sub-scales across the two samples of the present study are reported in Table 2.

#### Eating Behaviors

Cognitive and behavioral components of eating were measured by the short version of the Three-Factor Eating Questionnaire (TFEQ; Karlsson et al., 2000), comprising 18 items with a 1–4 response scale. All item responses are dichotomized and aggregated into three sub-scales, namely *emotional eating* (EE, three items; e.g., “When I feel anxious, I find myself eating”), *uncontrolled eating* (UE, nine items; e.g., “Sometimes when I start eating, I just can’t seem to stop”) and *cognitive restraint* (CR, six items; e.g., “I consciously hold back at meals in order not to gain weight”). This short-form was validated for the use in Spain (Jáuregui-Lobera et al., 2014), whereas for the Italian version we provided a list of items selected from a previously Italian validated long version (Melchionda et al., 2003) and tested the factor structure in the first Italian subsample of the present study. Reliability coefficients (i.e., Cronbach’s Alpha) of the three TFEQ’s subscales across the three samples are reported in Table 2.

### Statistical Analyses

#### Reliability and Descriptive Analyses

Reliability and descriptive analyses as well as MANOVAs were carried out through the SPSS software (Statistical Package for the Social Sciences - IBM, 2017) version 25. More specifically, three MANOVAs were carried out to explore possible differences across countries (Italy vs. Spain) in participants’ perfectionism, psychological distress, and eating behaviors.

#### Confirmatory Factor Analysis (CFA) of the Italian Version of the Tree Factor Questionnaire

In order to evaluate the factorial validity of the Italian 18-items Three-Factor Eating Questionnaire, using the data of the psychometric sample (*n* = 360), a confirmatory factor analysis (CFA) was carried out using M*plus* software version 7 (Muthén and Muthén, 2012) and the model parameters were estimated using the default robust weighted least squares (WLSMV) estimation method with theta parameterization to account for the categorical nature of the 4-point response scale (Brown, 2015). The adequacy of the CFA was ascertained using a variety of indices measuring the degree of fit between input data and model-based estimates. The literature indicates the following as good fit model indices: TLI (Tucker-Lewis Index) or CFI (Comparative Fit Index) values close to 0.95 (Hu and Bentler, 1999), RMSEA (Root Mean Square Error of Approximation) values below 0.08 (Marsh et al., 2004) and WRMR (Weighted Root Mean Squared Residuals) values below 0.95 (close to 1.0 reasonable fit; Yu, 2002); a chi-square/df ratio below or equal to 3 (Kline, 1998).

#### Measurement Invariance Analysis

Before testing the hypothesized model, the equivalence of the measurement model between countries (i.e., across the two main samples) is configured as a necessary condition for the comparison of psychological variables (Meredith, 1993). Although this was not the main purpose of our research, we considered it appropriate to conduct the measurement invariance separately for each of the three questionnaires we used for this study. In line with that, a series of multi-group confirmatory factor analyses (MGCFA) were modeled using maximum likelihood (ML) estimate, or the default robust weighted least squares estimate for ordinal data (WLSMV), via M*plus* software version 7 (Muthén and Muthén, 2012), as follows (e.g., Meredith, 1993): (1) configural invariance, in which each common factor is associated with the same items across countries; (2) metric invariance, in which the items presented invariant factor loadings, but item intercepts are freely estimated across countries; (3) scalar invariance, in which both factor loadings and item intercepts (or thresholds for ordinal data) are constrained to invariance. According to Meredith (1993), the average item and scale scores are comparable across the two countries when scalar invariance is supported (Tomás et al., 2014).

For the purpose of the current study, the nested models of measurement invariance were compared against the configural invariance model using the change in CFI, TLI and RMSEA. Literature (Widaman, 1985; Cheung and Rensvold, 2002) states that a Δ ≤ 0.01 for CFI and TLI, or Δ ≤ 0.015 for RMSEA (Chen, 2007), indicates a not significant worsening of the fit model. The chi-square difference tests using the DIFFTEST command, in which a non-significant value (*p* > 0.05) indicates good fit (Cheung and Rensvold, 2002), were also conducted to examine the ordinal data. However, if values exceeded these cut-offs criteria, partial invariance models were tested by releasing non-invariant parameters as stated by Byrne et al. (1989).

#### Multi-Group Structural Equation Model (SEM) Analysis

**FIGURE 1:**
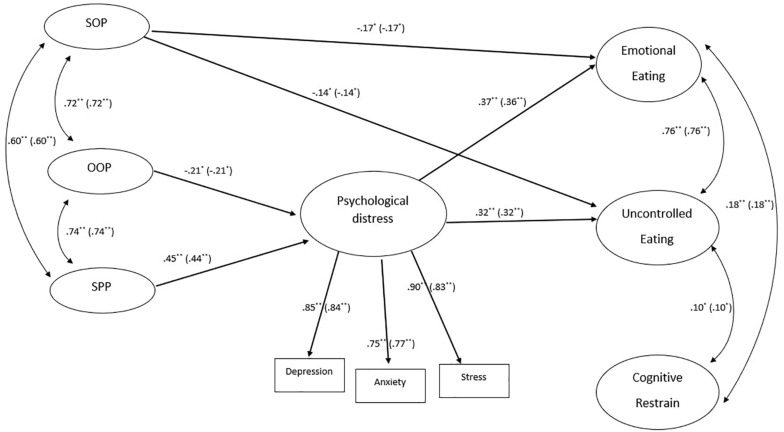
Tested model. The figure reported the standardized estimates both for Italian and Spanish (in parenthesis) samples that resulted statistically significant. The following not statistically significant paths were not depicted in figure for clarity: SOP→Psychological distress: β = –0.01, *p* = 0.84 (β = –0.01, *p* = 0.84); SOP→ Cognitive restrain: β = 0.12, *p* = 0.08 (β = 0.12, *p* = 0.08); OOP→Emotional eating: β = 0.14, *p* = 0.15 (β = 0.14, *p* = 0.15); OOP→Uncontrolled eating: β = 0.08, *p* = 0.36 (β = 0.08, *p* = 0.36); OOP→Cognitive restrain: β = –0.04, *p* = 0.66 (β = –0.04, *p* = 0.66); SPP→ Emotional eating: β = 0.02, *p* = 0.79 (β = 0.02, *p* = 0.79); SPP →Uncontrolled eating: β = 0.08, *p* = 0.30 (β = 0.08, *p* = 0.30); SPP →Cognitive restrain: β = 0.05, *p* = 0.51 (β = 0.05, *p* = 0.51); Psychological distress→Cognitive restrain: β = 0.08, *p* = 0.054 (β = 0.08, *p* = 0.054). Finally, the path linking the BMI with endogenous latent variables in the model were also freely estimated in multi-group SEM analysis, but they were not depicted in figure for clarity: BMI→Psychological distress: β = –0.02, *p* = 0.043 (β = –0.11, *p* < 0.001); BMI→Emotional Eating: β = 0.04; *p* < 0.001 (β = 0.19, *p* < 0.001); BMI→Uncontrolled Eating: β = 0.04, *p* < 0.001 (β = 0.19, *p* < 0.001); BMI→Cognitive Restrain: β = 0.03, *p* = 0.028 (β = 0.12, *p* = 0.026). In the figure the measurement section of the model was also omitted for clarity. However, all the information can be request to the corresponding authors. SOP, self-oriented perfectionism; OOP, other-oriented perfectionism; SPP, social-prescribed perfectionism. ^∗^ < 0.05; ^∗∗^ < 0.001.

Subsequently, we tested a model, across Italian (*n* = 465) and Spain (*n* = 352) main data, hypothesizing that perfectionism has direct and indirect effects (i.e., through its effect on psychological distress) upon eating behaviors (see Figure 1). Furthermore, the relations between these variables were tested controlling for the possible effect of participants’ body mass index (BMI) on the endogenous variables of the model (i.e., psychological distress and eating behaviors).

The hypothesized model was tested using a multi-group SEM analysis and the model parameters were estimated using the maximum likelihood (ML in M*plus*) estimation method. More specifically, the multi-group analysis was carried out in order to evaluate firstly the model measurement invariance parameters across the two countries (i.e., Model 1- Metric invariance) and, subsequently, the extent to which the model’s hypothesized relations held across them (i.e., Model 2-Covariances invariance and Model 3- Paths invariance).

In order to calculate the measurement indicators for the latent variables of the model, according to standard procedures for SEM analysis and following past studies (e.g., Lombardo et al., 2013), we used the three DASS’s subscales (i.e., depression, anxiety, and stress) as measurement indicators of the latent variable psychological distress. Furthermore, an item parceling procedure (Kim and Hagtvet, 2003) was used for the other latent variables, in line with previous studies (e.g., Lucidi et al., 2014, 2019; Mallia et al., 2015). Specifically, the item parcels for each of these latent variables (i.e., self-oriented perfectionism, other-oriented perfectionism, social prescribed perfectionism, emotional eating, uncontrolled eating, and cognitive restrain) were created by randomly grouping the items of each scale into three separate item sets (parcels) and by averaging the item scores within each set.

For the multi-group analysis, the three models introducing the invariances (i.e., metric, covariances, and paths) across the two countries were compared against a configural invariance model using cut-offs listed above for acceptable change in CFI, TLI, and RMSEA. Finally, the indirect effects of the model were examined using bootstrapped confidence interval estimates (95% confidence interval with 5000 bootstrap resamples).

## Results

### Reliability and Descriptive of the Key Measures of the Study

Overall, as reported in Table 2, all the key measures used in the present study showed acceptable internal consistency both in the Italian (Cronbach’s α ≥ 0.73) and in the Spanish (Cronbach’s α ≥ 0.75) main samples.

The MANOVAs results showed a significant multivariate effect of the country on the perfectionism [Wilks’s λ_(__3_,_813__)_ = 0.976; *p* < 0.001; $\eta_{\text{p}}^{2}$ = 0.024], on the psychological distress [Wilks’s λ_(__3_,_813__)_ = 0.947; *p* < 0.001; $\eta_{\text{p}}^{2}$ = 0.053] as well as on eating behaviors [Wilks’s λ_(__3_,_813__)_ = 0.976; *p* < 0.001;$\eta_{\text{p}}^{2}$ = 0.024]. However, considering the univariate effects, as reported in Table 2, emerged a significant difference across the two countries only on the socially prescribed perfectionism dimension, on depression and anxiety, and on uncontrolled eating. More specifically, the Italian respondents reported higher levels of socially prescribed perfectionism, higher levels of depression and anxiety, and lower levels of uncontrolled eating when compared with the Spanish respondents.

### Confirmatory Factor Analysis of the Italian Version of the Tree Factor Questionnaire

The CFA conducted on the Italian psychometric sample showed that the three-factor structure of the Tree Factor Questionnaire fits the data well [χ^2^_(__132__)_ = 365.892, *p* < 0.001; χ^2^e/df = 2.77, CFI = 0.96, TLI = 0.953, RMSEA = 0.070, 90% CI: from 0.062 to 0.079; WRMR = 1.23]. The standardized estimates of the factor loadings are reported in Appendix 1. All factor loadings of each of the three latent variables assessed by the questionnaire were statistically significant (*p* < 0.001) and were above 0.50.

### Measurement Invariance of the HFMPS, DASS, and TFEQ Measures Across Italian and Spanish Samples

We compared the fit of the three models to evaluate measurement invariance of the key measures of the study across the two countries. Scalar invariance, or at least partial scalar invariance, was achieved with several revisions necessary to satisfy the cut-offs criteria.

First, the HFMPS measure showed an acceptable fit of the model at the configural invariance step after correlating the residuals of item 2 and 1, and of item 14 and 11 [χ^2^_(__170__)_ = 617.134, *p* < 0.001; CFI = 0.91, TLI = 0.89, RMSEA = 0.08, 90% CI: from 0.073 to 0.087; SRMR = 0.073]. Changes in fit indices supported metric invariance (ΔCFI = 0.001, ΔTLI = 0.006, ΔRMSEA = 0.002), while they did not support scalar invariance initially (ΔCFI = 0.072, ΔTLI = 0.062, ΔRMSEA = 0.021). Inspection of the modification indices indicated that five item intercepts were non-invariant across countries (i.e., intercepts of item 12, 7, 11, 1, and 8). Thus, partial scalar invariance was supported after removing the equality constraints on these item intercepts (ΔCFI = 0.013, ΔTLI = 0.001, ΔRMSEA = 0.001) and testing the practical significance of differential item functioning (DIF) across countries. In the present study, the difference d was trivial (*d* < 0.20; Chan, 2000).

Second, the DASS measure displayed an acceptable fit of the model at the configural invariance step [χ^2^_(__372__)_ = 997.743; *p* < 0.001; CFI = 0.97, TLI = 0.97, RMSEA = 0.064, 90% CI: from 0.059 to 0.069; WRMR = 1.604]. Also, despite the significant chi-square difference, changes in the fit indices supported the metric invariance step [Δχ^2^_(__18__)_ = 36.625; *p* = 0.006; ΔCFI = 0.006; ΔTLI = 0.008; ΔRMSEA = 0.008], and the scalar invariance step [Δχ2_(__60__)_ = 594.922; *p* < 0.001; ΔCFI = 0.012; ΔTLI = 0.005; ΔRMSEA = 0.004].

Lastly, fit indices for the configural invariance step of the TFEQ measure fell within specified ranges [χ^2^_(__264__)_ = 725.688; *p* < 0.001; CFI = 0.97, TLI = 0.96, RMSEA = 0.065, 90% CI: from 0.060 to 0.071; WRMR = 1.752]. Moreover, changes in fit indices suggested that the fit of the metric invariance step was not significantly worse from that the configural model [Δχ^2^_(__15__)_ = 23.617; *p* = 0.0719; ΔCFI = 0.002, ΔTLI = 0.004, ΔRMSEA = 0.004], but the scalar invariance step showed a deterioration in fit: Δχ^2^_(__51__)_ = 501.063; *p* < 0.000; ΔCFI = 0.018, ΔTLI = 0.01, ΔRMSEA = 0.008. Inspection of the modification indices indicated that the main source of the misfit can be traced to one non-invariant threshold (item 1 threshold 2). Despite the chi-square difference was again significant [Δχ^2^_(__50__)_ = 358.191; *p* < 0.000], the changes in the fit indices compared to the configural model supported the partial scalar invariance by freeing this item threshold across countries (ΔCFI = 0.012, ΔTLI = 0.001, ΔRMSEA = 0.001).

### The Relationships Between Perfectionism, Psychological Distress, and Eating Behaviors Across Italian and Spanish Samples

Table 3 shows the results of the multi-group analysis, which was performed to verify the metric invariance, the covariances invariance and, finally, the path invariance of the hypothesized model across the two countries. In particular, the baseline model (i.e., M0 – Configural invariance) showed a good fit, attesting that the hypothesized model fit well both the Italian and the Spanish data. Furthermore, the comparisons between the models introducing three different constrains/invariances across the two samples showed not significant differences, since all the observed Δ CFIs are smaller than the recommended cut-offs (0.01). These results attested firstly that the factor loadings of the indicators used for each latent variable of the model resulted statistically equivalent across the two countries (i.e., metric invariance of the models). Additionally, the results attested also that the covariances between the latent variables of the model as well as the paths linking these variables resulted statistically equivalent across Italian and Spanish data (i.e., covariance and path invariance respectively). In particular, as reported in Figure 1, self-oriented perfectionism (i.e., SOP) showed a negative direct link both with emotional eating (β = –0.17) and with uncontrolled eating (β = −0.14). Other oriented perfectionism, instead, showed only indirect negative effects both on emotional eating (αβ = −0.082; 95% confidence interval: from −0.192 to −0.007) and uncontrolled eating (αβ = −0.072; 95% confidence interval: from −0.166 to −0.006). Conversely, social prescribed perfectionism showed only indirect positive effects both on emotional eating (αβ = 0.177; 95% confidence interval: from 0.109 to 0.300) and uncontrolled eating (αβ = 0.155; 95% confidence interval: from 0.094 to 0.257).

**TABLE 3:** Multi-group SEM: Models comparisons.

Model	Chi-square	df	CFI	TLI	RMSEA	SRMR	Chi-square/df	Comparison	Δ CFI	ΔTLI	ΔRMSEA
Model 0 (M0) – Configural invariance	894.195	384	0.944	0.933	0.057	0.054	2.32				
Model 1 (M1) – Metric invariance (i.e., factor Loadings)	977.527	405	0.937	0.928	0.056	0.051	2.41	M1 vs. M0	0.007	0.005	0.001
Model 2 (M2) – Covariances invariance	983.967	411	0.937	0.928	0.058	0.051	2.39	M2 vs. M0	0.007	0.005	0.001
Model 3- (M3) – Paths invariance	1041.644	430	0.933	0.928	0.058	0.057	2.42	M3 vs. M0	0.011	0.005	0.001

## Discussion

The present study investigated whether the psychological distress evaluated during the COVID-19 pandemic mediates the relationship between perfectionistic dimensions and problematic eating attitudes and behaviors in two samples drawn from the general population of Italy and Spain.

Findings showed that the psychological distress evaluated during the COVID-19 pandemic mediated the relationship between two perfectionistic dimensions and problematic eating behaviors in both the samples included. More specifically, results confirmed that psychological distress fully mediates the associations between the interpersonal aspects of perfectionism (i.e., SPP, OOP) and two of the three dysfunctional eating behaviors examined, namely emotional eating and uncontrolled eating. These results overlap with findings indicating that high perceived stress is associated with greater eating disorders symptoms (e.g., Klatzkin et al., 2019) as well as with components of perfectionistic concerns (i.e., SPP, OOP; Dunkley et al., 2016).

Path coefficients of each specific effect tested through the model indicated that participants with high SPP also show elevated psychological distress, that, in turn, is associated with greater levels of emotional eating and uncontrolled eating. This finding is consistent with previous results evidencing that maladaptive perfectionism positively predicts emotional eating through the mediation of stress (Wang and Li, 2017) and indicate that in the COVID-19 pandemic the link between perfectionism and eating symptoms is better explained by the mediation of the psychological distress. The role of SPP in predicting high psychological distress should be interpreted taking into account peculiarities of people with this perfectionistic aspect. Individuals with high SPP are prone to excessively concern over external expectations and pressures, and often engage in coping choices that did not match with the daily situational demands (Zhang, 2012). It is plausible that high SPP individuals may experience elevated stress in dealing with the provisions introduced by the governments in COVID-19 pandemic (i.e., social distancing), and that this stress is detrimental for eating behaviors. Moreover, previous evidence suggested that SPP is related to perceived loneliness and isolation (e.g., Harper et al., 2020). Recently, the importance of social connection is highlighted to mitigate negative psychological consequences of the COVID-19 pandemic (Tull et al., 2020). We speculate that the distress related to COVID-19 pandemic may facilitate people with high SPP in experiencing social disconnection, leading to eat more than usual (i.e., uncontrolled eating) and eating in response to emotional cues (i.e., emotional eating). This hypothesis is consistent with research on the association between eating behaviors and loneliness (Levine, 2012) that indicated the tendency to overeating as distracting from perceived social isolation and stress (Wansink and Payne, 2007).

Findings also showed that OOP negatively impacts psychological distress, that, in turn, positively predicts emotional eating and uncontrolled eating. These results evidence that demanding perfection from others (i.e., OOP) is associated with low psychological distress. Previous studies found a negative relationship between OOP and stress (Chang and Sanna, 2001), suggesting that, under certain circumstances, OOP could have adaptive effects (Hunter and O’Connor, 2003). Individuals with OOP are continuously concentrated on others’ performance thus it is possible that the tendency to direct the attentive focus away from self-scrutiny, in some circumstances, is beneficial (as cited in Hunter and O’Connor, 2003).

Additionally, results evidenced a not significant SOP- psychological distress association and a direct negative effect of SOP on emotional eating and uncontrolled eating. The lack of association between SOP and psychological distress is consistent with previous findings (e.g., Flett et al., 2016) and confirms the intrinsic functional nature of SOP. The adaptiveness of SOP is also suggested by the fact that participants with high SOP experienced low disturbed eating behaviors, and this relationship is independent of their perceived distress. This finding contradicts the well-established positive association between perfectionistic strivings (i.e., SOP) and eating symptoms (see Limburg et al., 2017). Instead, results suggest that SOP may play a protective role for the negative consequences of distress on emotional and uncontrolled eating. This conclusion is consistent with previous evidence showing that positive perfectionism (an aspect of perfectionism comparable to SOP) was related to low emotional eating levels (Wang and Li, 2017). Taken together, these observations suggest that adaptive perfectionists (i.e., those who have high SOP) may be less vulnerable to the tendency to overeat and to eat in response to negative emotions. It is plausible that the typical motivation to be flawless of individuals with high SOP makes them less prone to engage in uncontrolled eating and in eating in response to emotional cues in high-stress conditions. Empirical evidence showed that, among all the perfectionism dimensions, only SOP was significantly related to the rigid adherence to strict dietary rules (Brown et al., 2012), thus it is possible that during the public emergency of COVID-19 pandemic, high SOP may have lead people to rigidly interpret and adhere to guidelines for healthy eating.

No significant association was found for cognitive restrain. This result is inconsistent with results of previous studies evidencing a positive association between cognitive dietary restraint and perfectionism (e.g., Cain et al., 2008), as well as between this eating behavior and levels of perceived stress (e.g., McLean and Barr, 2003). Social isolation measures introduced for the containment of the COVID-19 pandemic (including stay-at-home mandates) may make it difficult for people to restrict eating with the intention to lose or maintain weight. Staying at home could facilitate the engaging in unhealthy behaviors such as overeating (as cited in Buenaventura et al., 2020) rather than cognitive restrain due to the easy access to food reserves (e.g., Rodgers et al., 2020).

Several limitations of the present study should be acknowledged before concluding. First, its cross-sectional nature limited causal inferences. Further longitudinal studies are needed to examine whether perfectionistic dimensions prospectively predict changes in eating behaviors, and if this association would be mediated by psychological distress. Second, the mere use of self-report questionnaires may be subject to social desirability effects and recall bias. More specifically, future studies should control for the effect of social desirability as previous evidence showed positive associations between the motivation to distort one’s responses in a favorable direction (i.e., social desirability) and both perfectionism and psychological distress (e.g., Lopez et al., 2006; Kung and Chan, 2014). Further studies should include a measure of perceived social isolation in order to analyze the extent to which each aspect of perfectionism could be related to the experience of loneliness during COVID-19 pandemic. Moreover, the current study should be replicated in other cultures to strengthen the generalizability of the results.

Despite these limitations, the present study provides further evidence for the roles of each perfectionism dimension in eating behaviors by considering the mediation role of psychological distress. Strengths of the present work include the use of multi-group SEM as a rigorous way to examine the degrees of goodness-of-fit for the proposed model and to simultaneously compare parameters across the two groups (Italian and Spanish). The multi-group SEM has been regarded as a powerful model and recommended in cross-cultural research especially when computing pairwise comparison between countries (Feskens and Hox, 2011). Moreover, the establishment of measurement invariance of all the scales used strongly supported the generality of the model and warranted comparisons between the two cultural groups studied.

In summary, results of the present research suggest that the OOP and SPP dimensions resulted to indirectly predict emotional and uncontrolled eating, whereas non-significant mediation result was found for SOP. Instead, SOP was found to negatively predict eating behaviors, supporting the adaptive nature of SOP in relation to emotional eating and uncontrolled eating and suggesting its potential protective role in conditions of stress and social isolation. These findings have theoretical implications for the perfectionism and eating symptoms literature. For example, the negative effect of SOP on eating symptoms may be the consequence of controlling for the common variance shared with other perfectionistic dimensions. Previous studies demonstrate that when the covariance between perfectionistic strivings (e.g., SOP) and perfectionistic concerns (e.g., SPP) was controlled for, perfectionistic strivings show negative associations with maladaptive outcomes (e.g., anxiety; Stoeber et al., 2007). This mechanism may also pertain eating disorders (EDs) symptoms, as one past study demonstrated (Minarik and Ahrens, 1996). Further research should shed light on these processes by employing sophisticated statistical methods to more deeply explore the simultaneous effects of SOP, SPP, and OOP on problematic eating behaviors.

Findings suggest some clinical implications for future research and interventions aimed at reducing problematic eating behaviors. A growing literature supports that programs targeting perfectionism may be an effective treatment for EDs (e.g., Wilksch et al., 2008; Goldstein et al., 2014). Generally, these interventions address multiple components of perfectionism (e.g., concern over mistakes, personal standards; Wilksch et al., 2008) and result in a reduction of EDs symptoms (e.g., shape and weight concerns, Wilksch et al., 2008; drive for thinness, Levinson et al., 2017). In the present investigation, the interpersonal perfectionistic aspects (i.e., SPP, OOP) showed significant indirect associations with eating symptoms, suggesting that intervention protocols for individuals suffering from EDs should especially address these forms of perfectionism, as previous studies highlighted (e.g., Reilly et al., 2016). EDs treatment targeting perfectionism typically emphasizes changes in the patient’s scheme for self-evaluation and includes cognitive-behavioral methods to address personal standards and self-criticism (see Egan et al., 2014 for further details). Results of this study may imply that future prevention and treatment for problematic eating behaviors should mainly aim on the reduction of aspects related to SPP (e.g., fear of failure) and OOP (e.g., excessive other-criticism) rather than aspects of SOP, although evidence on the efficacy of EDs treatment designed to decrease SOP is available in the literature (e.g., Lethbridge et al., 2011). Instead, the present findings highlighted the protective role of SOP in the development and maintenance of problematic eating behaviors. Whereas the reduction of perfectionistic concerns (e.g., SPP) through the use of specific techniques (e.g., self- compassion strategies) should be recommended, the drive to excel related to SOP could be re-addressed to enhancing motivation for change and to serve recovery in EDs treatment (Wagner and Vitousek, 2019). On the other hand, clinicians should pay special attention to the reduction of perceived distress. More specifically, treating perfectionistic concerns may result in a relative reduction of patients’ psychological distress, which, in turn, would decrease the likelihood to engage in emotional eating and uncontrolled eating behaviors. Further investigations of specific treatment strategies targeting these processes are required.

## Data Availability Statement

The raw data supporting the conclusions of this article will be made available by the authors, without undue reservation.

## Ethics Statement

The studies involving human participants were reviewed and approved by Institutional Review Board – Department of Psychology, Sapienza University of Rome. The participants provided their written informed consent to participate in this study.

## Author Contributions

All the authors contributed to the study conception and design, and commented on previous versions of the manuscript. MV and ADM performed the material preparation, literature research, and data collection. ADM performed the statistical analyses with the supervision of LM. MV wrote the first draft of the manuscript. CL and LM read, corrected, and approved the final manuscript.

## Conflict of Interest

The authors declare that the research was conducted in the absence of any commercial or financial relationships that could be construed as a potential conflict of interest. The reviewer IT declared a past collaboration with several of the authors, ADM and LM, to the handling editor.

## Footnotes

1 https://www.cdc.gov/coronavirus/2019-ncov/prevent-getting-sick/social-distancing.html

2 We preliminary evaluated the factorial structure of the Spanish short version of the HMPS on the main Spanish sample using a Confirmatory Factor Analysis. The results showed that the three-factor structure of the scale fits the data almost well [χ^2^_(__87__)_ = 401.013, *p* < 0.001; CFI = 0.87, RMSEA = 0.10, 90% CI: from 0.091 to 0.111; SRMR = 0.095]. The standardized estimates of the factor loadings and other details can be provided on request by the first author. However, the factor loadings of each of the three latent variables assessed by the questionnaire were statistically significant (*p* < 0.001) and were above 0.43.

## Appendix 1

**Table:** Standardized factor loadings of the short version of the Three Factor Questionnaire.

	Emotional eating	Uncontrolled eating	Cognitive restrain
Item 3	0.845*		
Item 6	0.934*		
Item 10	0.814*		
Item 1		0.660*	
Item 4		0.835*	
Item 5		0.726*	
Item 7		0.672*	
Item 8		0.831*	
Item 9		0.800*	
Item 13		0.774*	
Item 14		0.609*	
Item 17		0.716*	
Item 2			0.762*
Item 11			0.844*
Item 12			0.730*
Item 15			0.522*
Item 16			0.783*
Item 18			0.690*

**p level < 0.001.*
